# The Transdiagnostic Intervention for Sleep and Circadian Dysfunction (TranS-C) for serious mental illness in community mental health part 3: study protocol to evaluate sustainment in a hybrid type 2 effectiveness-implementation cluster-randomized trial

**DOI:** 10.1186/s13063-023-07900-1

**Published:** 2024-01-15

**Authors:** Laurel D. Sarfan, Emma R. Agnew, Marlen Diaz, Ashby Cogan, Julia M. Spencer, Rafael Esteva Hache, Shannon Wiltsey Stirman, Cara C. Lewis, Amy M. Kilbourne, Allison G. Harvey

**Affiliations:** 1 Department of Psychology, University of California, 2121 Berkeley Way, Berkeley, CA 94720 USA; 2 Dissemination and Training Division, National Center for PTSD, Palo Alto, CA USA; 3 Department of Psychiatry and Behavioral Sciences, Stanford University, Palo Alto, USA; 4 Kaiser Permanente Washington Health Research Institute, Seattle, USA; 5 Quality Enhancement Research Initiative, U.S. Department of Veterans Affairs, Washington, D.C., USA; 6 Department of Learning Health Science, University of Michigan Medical School, Ann Arbor, MI USA

**Keywords:** Sustainment, Implementation, Train-the-trainer, Adaptation, Community mental health, Transdiagnostic, Sleep, Circadian, Serious mental illness, TranS-C

## Abstract

**Background:**

Although research on the implementation of evidence-based psychological treatments (EBPTs) has advanced rapidly, research on the sustainment of implemented EBPTs remains limited. This is concerning, given that EBPT activities and benefits regularly decline post-implementation. To advance research on sustainment, the present protocol focuses on the third and final phase—the Sustainment Phase—of a hybrid type 2 cluster-randomized controlled trial investigating the implementation and sustainment of the Transdiagnostic Intervention for Sleep and Circadian Dysfunction (TranS-C) for patients with serious mental illness and sleep and circadian problems in community mental health centers (CMHCs). Prior to the first two phases of the trial—the Implementation Phase and Train-the-Trainer Phase—TranS-C was adapted to fit the CMHC context. Then, 10 CMHCs were cluster-randomized to implement Standard or Adapted TranS-C via facilitation and train-the-trainer. The primary goal of the Sustainment Phase is to investigate whether adapting TranS-C to fit the CMHC context predicts improved sustainment outcomes.

**Methods:**

Data collection for the Sustainment Phase will commence at least three months after implementation efforts in partnering CMHCs have ended and may continue for up to one year. CMHC providers will be recruited to complete surveys (*N* = 154) and a semi-structured interview (*N* = 40) on sustainment outcomes and mechanisms. Aim 1 is to report the sustainment outcomes of TranS-C. Aim 2 is to evaluate whether manipulating EBPT fit to context (i.e., Standard versus Adapted TranS-C) predicts sustainment outcomes. Aim 3 is to test whether provider perceptions of fit mediate the relation between treatment condition (i.e., Standard versus Adapted TranS-C) and sustainment outcomes. Mixed methods will be used to analyze the data.

**Discussion:**

The present study seeks to advance our understanding of sustainment predictors, mechanisms, and outcomes by investigating (a) whether the implementation strategy of adapting an EBPT (i.e., TranS-C) to the CMHC context predicts improved sustainment outcomes and (b) whether this relation is mediated by improved provider perceptions of treatment fit. Together, the findings may help inform more precise implementation efforts that contribute to lasting change.

**Trial registration:**

ClinicalTrials.gov identifier: NCT05956678. Registered on July 21, 2023.

**Supplementary Information:**

The online version contains supplementary material available at 10.1186/s13063-023-07900-1.

The gap between research and practice is widely recognized [1]. There is a long delay between the development of evidence-based psychological treatments (EBPTs) and translation of EBPTs into practice [2]. Moreover, only a fraction of EBPT research is translated into routine practice settings [3]. In response, implementation science has emerged. The National Institutes of Health define implementation as “the use of strategies to adopt and integrate evidence-based health interventions ...within specific settings” [4]. Although some studies have produced mixed findings [5], there is compelling evidence that implementation efforts yield promising results: EBPTs can be implemented, and implemented EBPTs can improve patient outcomes [6, 7].

While research on implementation has advanced rapidly, research on *sustainment* of implemented EBPTs remains limited [8–11]. According to Shediac-Rizkallah and Bone’s widely used framework [12], sustainment is defined as continued (a) activities, (b) benefits, and (c) capacity related to an intervention after implementation efforts have ended. Leading implementation scientists have labeled the dearth of sustainment research as “one of the most significant translational research problems of our time” (p. 2) [9]. Sustainment research is critical for several reasons. First, after implementation supports have ended, EBPT activities and benefits regularly decline—a phenomenon known as “voltage drop” [8, 13]. Second, implementation efforts often require substantial investment from funders, researchers, and community stakeholders; thus, successful sustainment can help ensure these investments have yielded lasting returns [8]. Third, from a clinical lens, evaluating sustainment is essential to ensure that patients continue to receive optimal care post-implementation. Fourth, many leading implementation science frameworks characterize sustainment as a vital stage of implementation science, but the empirical findings to support these frameworks lag behind [14]. Fifth, the dearth of sustainment research means that *predictors and mechanisms* of EBPT sustainment are largely unknown.

Leaders in implementation science have highlighted the importance of testing predictors and mechanisms to improve our understanding of how and why sustainment is successful, which, in turn, can inform more targeted and efficient implementation and sustainment efforts [15, 16]. The few empirical studies that have identified significant predictors and mechanisms of sustainment hold enormous potential because they pinpoint *targets* to maximize sustainment outcomes. In particular, findings from a handful of studies suggest that sustainment outcomes are predicted by treatment “fit” within a given context [17, 18] and provider perceptions of treatment [18, 19]. This aligns with several influential frameworks that have identified provider perceptions of treatment fit as key to implementation and sustainment success [20–22]. Putting these pieces together, treatment fit and provider perceptions of treatment represent potential targets that could improve sustainment outcomes. However, to our knowledge, no prior research has taken the next step of testing whether *manipulating* fit predicts sustainment mechanisms (e.g., provider perceptions of treatment fit) or sustainment outcomes. Together, the protocol for the study herein aims to advance the field’s understanding of sustainment by evaluating sustainment (a) outcomes (i.e., continued activities, benefits, and capacity), (b) predictors (i.e., manipulating fit to context), and (c) mechanisms (i.e., provider perceptions of fit) of an EBPT implemented in routine practice settings.

This study is the third phase of a three-phase cluster-randomized controlled trial. Broadly, the trial is focused on the Transdiagnostic Intervention for Sleep and Circadian Dysfunction (TranS-C) delivered to patients diagnosed with serious mental illness (SMI) in community mental health centers (CMHCs) across California in the USA. TranS-C is a modular, psychosocial treatment that is based on the Sleep Health Framework [23]. It was developed in light of the following three lines of research that support sleep and circadian problems as transdiagnostic contributors to SMI. First, sleep and circadian problems (e.g., insomnia, hypersomnia, evening circadian preference) are highly comorbid with and predict a range of SMI diagnoses (e.g., depression, substance use, anxiety, psychosis) [24–26]. Second, common cognitive, behavioral, and neurobiological mechanisms (e.g., rumination, avoidance, and arousal) may predict and maintain both SMI and sleep and circadian problems [27, 28]. Third, treatments that address sleep and circadian problems have been concurrently associated with improvements in mental health symptoms [29–31].

Initial efficacy data for TranS-C delivered to individuals diagnosed with SMI in a CMHC setting are strong. A study conducted in a CMHC found that TranS-C was associated with reductions in sleep-related problems, functional impairment, and psychiatric symptoms relative to usual care followed by delayed treatment with TranS-C [29]. However, in this prior study, the therapists delivering TranS-C were employed by the research team, not the CMHC. Thus, to gather preliminary data on CMHC providers’ perceptions of TranS-C, Gumport and colleagues interviewed CMHC staff about their perceptions of TranS-C [32]. Themes from these interviews revealed provider perceptions that (a) the CMHC context is substantively different from the academic context in which many EBPTs, including TranS-C, are developed, (b) EBPTs need to be adapted to the CMHC context, and (c) providers have limited time to address their patients’ needs. Based on this feedback from providers—as well as pilot data, patient feedback, and theories guiding TranS-C and treatment adaptation—“Adapted TranS-C” was developed [33]. Relative to the original version of TranS-C (i.e., “Standard TranS-C”), Adapted TranS-C consists of fewer modules, shorter sessions, and briefer training (see the “Method” section and Table 1 for comparison) [33].

**Table 1:** Comparison of Standard and Adapted TranS-C trainings, sessions, and modules

	Standard TranS-C	Adapted TranS-C
Training	6–8 h	4 h
Session length	50 min	20 min
Number of sessions	8	4
TranS-C modules		
*Cross-cutting*		
Functional analysis	Yes	Yes
Education	Yes	Yes
Motivational enhancement	Yes	Yes
Goal setting	Yes	Yes
*Core modules*		
Regular sleep-wake times	Yes	Yes
Wind-down routine	Yes	Yes
Wake-up routine	Yes	Yes
Improving daytime functioning	Yes	Yes
Unhelpful beliefs about sleep	Yes	No
Maintaining your gains	Yes	Yes
*Optional modules*		
Reducing sleep-related worry	Yes	Yes
Improving sleep efficiency	Yes	No
Reducing time in bed	Yes	No
Delayed or advanced phase	Yes	No
CPAP machine and exposure	Yes	No
Negotiating complicated environments	Yes	No
Reducing nightmares	Yes	No

The core modules of Regular Sleep-Wake Times, Wind-Down Routine, and Wake-Up Routine are delivered as one core module in Standard TranS-C and three core modules in Adapted TranS-C. “Yes” indicates that the module is included in the given version of TranS-C. “No” indicates that the module is not included in the given version of TranS-C

Building on this past research, the overall goal of the three-phase randomized controlled superiority trial is to compare the implementation and effectiveness outcomes of Adapted TranS-C relative to Standard TranS-C, when delivered by CMHC providers to patients with sleep and circadian problems and SMI. In phase 1 of the trial, the Implementation Phase, sites were cluster randomized by county to Standard TranS-C or Adapted TranS-C with 1:1 allocation, and external facilitation was used to help implement TranS-C in partnering CMHCs [33]. In phase 2, the Train-the-Trainer Phase, CMHC providers were trained by facilitators to train and supervise their colleagues in the delivery of TranS-C [34]. See below for more details.

The present protocol focuses on phase 3 of the trial, the Sustainment Phase. The big picture question of the Sustainment Phase is the following: to what extent is TranS-C sustained after implementation activities have ended? More specifically, aim 1 of the present study is to report the sustainment outcomes of TranS-C after implementation support has ended. Following Shediac-Rizkallah and Bone’s framework [12], continued (a) activities, (b) benefits, and (c) capacity related to TranS-C will be reported. Aim 2 is to evaluate whether manipulating fit to context predicts sustainment outcomes. It is hypothesized that providers in Adapted TranS-C will report better sustainment outcomes (i.e., activities, benefits, and capacity) relative to Standard TranS-C. Aim 3 is to test whether provider perceptions of fit mediate the relation between treatment condition (Standard versus Adapted TranS-C) and sustainment outcomes. It is hypothesized that Adapted TranS-C, compared to Standard TranS-C, will predict better sustainment outcomes (i.e., activities, benefits, and capacity) indirectly through better provider perceptions of fit.

## Method

**Fig. 1:**
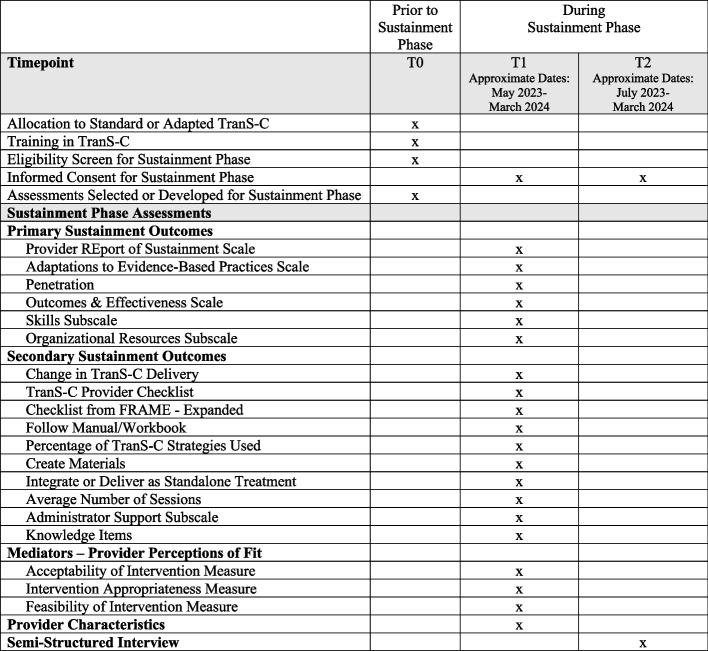
SPIRIT diagram of enrollment, interventions, and assessments for the Sustainment Phase. Allocation to Adapted or Standard TranS-C occurred at the county level and prior to enrollment of any participants in that county (i.e., patients or providers). For the Sustainment Phase, the assessments (i.e., surveys and semi-structured interview) are collected after the Implementation Phase, Train-the-Trainer Phase, and 3-month Sustainment Period. Providers are first recruited for the surveys (i.e., T1), then recruited for the interview (i.e., T2). TranS-C, Transdiagnostic Intervention for Sleep and Circadian Dysfunction. FRAME, Framework for Reporting Adaptations and Modifications**—**Expanded

This study was preregistered on ClinicalTrials.gov (identifier: NCT05956678) and received approval from the Committee for the Protection of Human Subjects at the University of California, Berkeley. Any protocol changes will be submitted or reported to ClinicalTrials.gov, the Committee for the Protection of Human Subjects, the participating CMHCs, and in appropriate publications. If there are too many findings to reasonably interpret in one paper, findings may be separated into two or more papers. The present protocol used the SPIRIT reporting guidelines (see SPIRIT checklist in Additional file 1 and SPIRIT diagram in Fig. 1) [35].

### Participants

Participants in the Sustainment Phase are providers from CMHCs included in the Implementation or Train-the-Trainer Phases, for which the inclusion criteria were (1) provision of publicly funded adult mental health outpatient services and (2) support from CMHC leadership.

The inclusion criteria for providers in the present study are as follows: (1) employed, able to deliver, or have delivered patient-facing services to patients within a CMHC^1^; (2) have attended a TranS-C training; (3) CMHC site of employment has been in the Sustainment Phase for at least 3 months; and (4) volunteer to participate and formally consent to participate. Note that CMHCs preferred to determine which providers were eligible to receive TranS-C training at each site (e.g., case managers, nurses, psychiatrists), because this aligns with their real-world practice.

It may be helpful to note that, in the Implementation and Train-the-Trainer Phases, providers were trained to deliver TranS-C to adult patients who met criteria for SMI and exhibited a sleep or circadian problem. However, given the focus on provider-level data in the Sustainment Phase as well as feasibility and resource constraints, patient data are not assessed in the present study (see also the “Discussion” section).

### Recruitment

#### Community mental health centers

Building the CMHC network that forms the basis for this study began in August 2013 with outreach by the principal investigator of the three-phase trial (AGH). Originally, eight counties—generally consisting of three to 10 CMHC sites—agreed to participate in the Implementation Phase. At various stages of the study, recruitment of new counties and new CMHC sites continued in order to maximize provider and patient sample size goals for the Implementation and Train-the-Trainer Phases. For instance, two additional counties (i.e., Lake and Kings counties) were recruited to account for fluctuations in engagement. In general, sites were selected based on interest from partners and to balance diversity (e.g., racial/ethnic backgrounds of patients served; urban vs. rural locations) with feasibility (e.g., manageable driving distance from University of California Berkeley). Sites in the following ten counties in California, USA, are included in the Sustainment Phase recruitment efforts: Alameda, Contra Costa, Kings, Lake, Monterey, Placer, Santa Barbara, Santa Cruz, Solano, and Santa Clara. Note that sites in San Lois Obispo are also participating but are operating as part of Monterey County. A list of the study sites can be found on ClinicalTrials.gov (NCT05956678).

#### Providers

**Fig. 2:**
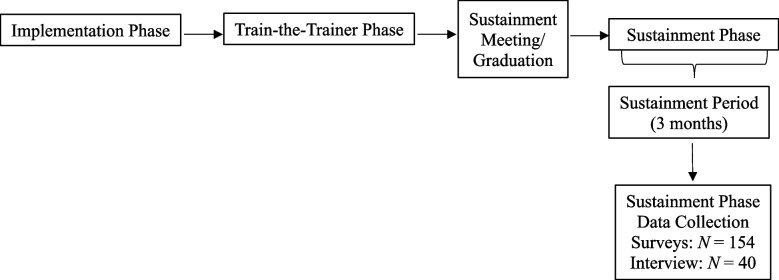
Trial procedure

For the Sustainment Phase, eligible providers are contacted by email and invited to complete a survey and interview about TranS-C. See Figs. 1 and 2 for duration of recruitment periods. Recruitment rates are regularly monitored by the first author. Providers are compensated for their time depending on local policies for receiving payment for research-related activities (e.g., compensated with gift card or treatment-related book).

### Interventions

As mentioned above, two variations of TranS-C are tested in this trial: Standard TranS-C and Adapted TranS-C (see Table 1 for comparison). Both are delivered alongside the usual care offered by each CMHC. In the CMHCs, usual care consists of working with a service provider (e.g., psychologist, case manager, occupational therapist, psychiatrist, nurse practitioner) who provides direct mental health support from within their scope of practice. The patient might also be referred by that provider for other services as needed (e.g., healthcare, housing support, nutrition, vocational specialists, or peer advocacy). Occasionally, patients receive treatment from interdisciplinary or residential teams, meaning their services are coordinated across multiple service providers. The TranS-C treatment conditions, along with the adaptation process for Adapted TranS-C, are described below. The modules that make up Standard and Adapted TranS-C are listed in Table 1 and described in detail elsewhere [33]. While the ordering of modules is broadly suggestive of the order of completion, providers are trained to be sensitive to the differences between patients as to which processes are key to maintaining their distress and to address these processes at an earlier stage of treatment. Although most providers deliver TranS-C via individual sessions, some choose to deliver it in a group setting. Of note, TranS-C was originally developed in English, then translated into Spanish and offered by Spanish-speaking providers during the Implementation Phase to expand access.

#### Standard TranS-C

Standard TranS-C is delivered via eight, 50-min weekly sessions and comprised of four *cross-cutting modules* featured in every session, four *core modules* delivered to most patients, and seven *optional modules* that are used based on clinical presentation, treatment goals, and provider case conceptualization [36]. Initial training for providers in the Standard TranS-C condition consists of a 1-day workshop (i.e., 6 to 8 h) or two, 3-h training blocks, based on CMHC preferences.

#### Adapted TranS-C

Adapted TranS-C is delivered via four, 20-min weekly sessions and comprised of the same *cross-cutting* and *core modules* as in Standard TranS-C (with one exception in the core modules, see Table 1). Additionally, there is one *optional module* that can be integrated with the core modules, based on clinical presentation, treatment goals, and provider case conceptualization. Training for the Adapted TranS-C condition consists of four, 1-h workshops or two, 2-h workshops, based on CMHC preferences.

The process of adapting TranS-C was grounded in theory, data, and stakeholder input. As the overarching guide for the adaptation process, the Replicating Effective Programs (REP) framework [13] was used. To summarize, the following were considered for REP: the need and evidence for TranS-C [29], data from interviews with stakeholders [32, 37], a pilot study of Adapted TranS-C (unpublished data), and TranS-C’s theoretical underpinnings and mechanisms of action [23, 36]. Additionally, following adaptation and treatment development frameworks, Adapted TranS-C was designed for a broad range of patient and implementation characteristics (e.g., symptom severity; CMHC resources) [38]. Additional details about the adaptation process are described elsewhere [33].

### Facilitation: Implementation, Train-the-Trainer, and Sustainment Phases

During the Implementation and Train-the-Trainer Phases of the three-phase trial, facilitation was used as the core implementation strategy, based on the Enhanced-REP framework [39] and promising evidence [40, 41]. Specifically, each CMHC received direct support from a lead facilitator (ERA)—who also served as the expert TranS-C trainer—as well as a team of trained facilitators, all of whom were employed by the research team and supervised by the principal investigator (AGH).

In the Implementation Phase, the external facilitators supported implementation of TranS-C in participating CMHCs through a range of activities, including leading TranS-C trainings, distributing TranS-C manuals and other educational materials, holding weekly TranS-C supervision and as-needed consultation, problem solving administrative barriers such as negotiating productivity requirements and ensuring that TranS-C activities counted toward Continuing Education Unit credits (CEUs), offering sleep treatment certification, and collaborating with leadership, key providers, and site champions. Additional details about the Implementation Phase are reported elsewhere [33].

The facilitation team transitioned CMHC sites to the Train-the-Trainer Phase on a rolling basis. The first site was transitioned to the Train-the-Trainer Phase in December 2020, and all sites were transitioned by December 2022 [33, 34]. During the Train-the-Trainer Phase, the facilitators engaged in the following: recruiting, training, and providing consultation for local CMHC trainers; recruiting and enrolling providers and patients; holding as-needed consultation for TranS-C providers; offering certification in sleep treatment and sleep training; processing CEUs; and organizing regular meetings with CMHC leadership to problem-solve barriers. Additionally, as CMHC providers and trainers gained mastery and independence, the facilitation team gradually transferred the following responsibilities to them: TranS-C trainings, clinical supervision, presentations on advanced topics to other providers, and cross-county consultation among trainers (termed the “Sleep Expert Network”). Additional details about the Train-the-Trainer Phase are reported elsewhere [34].

Sites were transitioned from the Train-the-Trainer Phase to the Sustainment Phase between January 1, 2023, and June 1, 2023. When transitioning CMHC sites to the Sustainment Phase, facilitators considered several factors. These factors included patient and provider recruitment, CMHCs’ established procedures to sustain TranS-C (e.g., scheduled TranS-C trainings on the calendar), CMHC provider and trainer mastery, and support from CMHC leadership. For each site, facilitators drafted individually tailored sustainment plans, which consisted of detailed checklists to help leadership, providers, and trainers establish systems to support continued delivery and training in TranS-C in the following three domains: (1) providing patients with TranS-C, (2) training and supporting TranS-C providers, and (3) identifying, training, and supporting TranS-C trainers. When a site completed most or all items on their sustainment plan, the facilitators held a sustainment meeting with CMHC leadership, providers, and/or trainers to answer any final questions. After this meeting, the site officially graduated to the “Sustainment Period” during which the site received minimal facilitation support for 3 months. Note that 3 months were selected for the Sustainment Period following research precedent that clinics may be at risk of sustainment failure as early as 3 months after implementation efforts have ended [42]. Following the Sustainment Period, providers are recruited for Sustainment Phase data collection (see Fig. 2), which began in May 2023. Depending on recruitment progress, sustainment data collection may continue for up to approximately 1 year (i.e., through March 2024).

Here, we note that the initial plan was to withdraw *all* support during the Sustainment Phase. However, upon deliberation and consultation with CMHC partners and experts in clinical service implementation and delivery, we decided that facilitators could continue (a) treatment-related assistance that is typically sought from outside experts or entities in clinical settings and (b) minimal background support. The treatment-related assistance consists of: provider-initiated informal consultation with facilitators (e.g., akin to “curbside consultation” with external experts); organizing CEUs and sleep coaching certification (e.g., akin to an outside institution offering CEUs or EBPT certification); and sending workbooks and manuals to counties as needed but no more than once per month (e.g., akin to an outside organization offering limited free treatment-related resources). The minimal background support consists of: gathering recordings of TranS-C trainings led by CMHC trainers and sitting in on presentations on advanced sleep-related topics and Sleep Expert Network meetings. This ongoing minimal background support is provided for two reasons: (1) to enable continued data collection (e.g., to compare training techniques of expert facilitators relative to CMHC trainers) and (2) to preserve community partnerships (e.g., sitting in on CMHC providers’ presentations to help them feel supported and encouraged).

### Measures

The measures described below are organized by the three domains of Shediac-Rizkallah and Bone’s sustainment framework (i.e., activities, benefits, and capacity [12]) followed by the proposed mechanism (i.e., provider perceptions of fit). Activities are operationalized as providers’ delivery, adaptations, and routinization of TranS-C in clinical practice [12]. Benefits are operationalized as providers’ assessment of TranS-C’s health benefits for patients [12]. Capacity is operationalized as providers’ knowledge, skills, and resources to deliver TranS-C [12]. Provider perceptions of fit are operationalized as TranS-C’s perceived acceptability, appropriateness, and feasibility [43–45].

Only measures that will be analyzed for the primary aims of the Sustainment Phase are reported below. One additional, novel measure was preregistered on ClinicalTrials.gov but not described below (i.e., Adaptations in Response to Cultural Backgrounds of Patients). The authors plan to conduct an evaluation of the measure’s psychometric properties, which, if substantiated, will support its utility for future studies. All measures were delivered at only one timepoint during the Sustainment Phase (see Fig. 1), and thus, the metric of interest is the value at this timepoint (see below for the specific measure, domain, and method of aggregation for each outcome). Note that for *all* measures below, providers are asked to consider *only* the time since their CMHC graduated to the Sustainment Phase. For all relevant questions, providers are offered a “not applicable” option if they have not delivered TranS-C during the Sustainment Phase. For some measures, the language was modified slightly from the original measure to increase accessibility and relevance for providers (e.g., changing “intervention” to “sleep treatment”).

#### Provider characteristics

Providers are asked to report their degree, theoretical orientation, age, sex assigned at birth, gender, ethnicity, and race. For categorical variables (e.g., degree), proportions expressed as a percentage will be reported. For continuous variables (e.g., age), average values will be reported.

#### Activities: delivery, adaptations, and routinization

##### Primary outcomes

The Provider REport of Sustainment Scale assesses providers’ continued delivery of TranS-C [46]. It was designed as a brief, pragmatic measure for direct service providers to report their continued use of a given evidence-based practice. The measure consists of three items that are rated on a scale from 0 (not at all) to 4 (to a very great extent), with the mean score calculated and higher scores indicating more sustainment. This measure has demonstrated acceptable internal consistency reliability (Cronbach’s alpha = 0.95; McDonald’s omega = 0.95) and construct validity within a similar sample of providers [46].

The Adaptations to Evidence-Based Practices Scale [47] assesses provider adaptations to treatment. Providers are asked to rate six items using a 4-point Likert scale from 1 (not at all) to 4 (very great extent)^2^. Each item assesses the extent to which providers have made a specific type of adaptation during the Sustainment Phase (e.g., modifying presentation, shortening or condensing pacing, removing or skipping components). The mean score will be calculated, with higher scores indicating greater use of adaptations. This measure has demonstrated acceptable reliability and construct validity within a similar sample of providers [47].

Penetration is assessed following a widely used definition and formula [48]. Specifically, providers are asked to report (a) how many of their patients have had sleep problems during the Sustainment Phase and (b) the number of those patients with whom the provider has used TranS-C during the Sustainment Phase. Then, “b” is divided by “a,” with higher scores indicating more penetration, expressed as a proportion. The mean proportion will be reported. In a review of implementation science measures, this formula was found to have excellent usability [49].

##### Secondary outcomes

As a proxy for change in TranS-C delivery, providers are asked whether they are using TranS-C “more,” “about the same,” or “less” relative to before the Sustainment Phase. As another secondary measure of delivery, providers complete the TranS-C Provider Checklist (modified for providers in the Adapted TranS-C condition to include only the relevant modules) [50]. On this measure, providers identify the cross-cutting, core, and optional modules they have delivered during the Sustainment Phase. The TranS-C Provider Checklist has demonstrated acceptable internal consistency (Cronbach’s alpha = 0.74, mean interitem correlation *ρ* = 0.16 ) and convergent validity among university-hired therapists delivering TranS-C at a CMHC [50]. 3 These two secondary outcomes will be reported as frequencies.

As a secondary measure of adaptations, providers complete a checklist from the coding manual for the Framework for Reporting Adaptations and Modifications—Expanded [51]. On this checklist, they indicate whether they have made any adaptations to TranS-C during the Sustainment Phase based on patient characteristics, such as race or ethnicity, gender identity, first/spoken language, literacy and education level, comorbidity/multimorbidity, and motivation and readiness. This outcome will be reported as frequencies (i.e., number of providers who reported making adaptations per each patient characteristic). As additional secondary measures of adaptations, providers are asked (a) to rate the extent to which they follow the TranS-C provider manual and patient workbook on a scale from 0% (not at all) to 100% (always/completely) [mean scores will be reported]; (b) to estimate the percentage of TranS-C strategies they use on average when delivering TranS-C to their patients on a scale from 0% (no strategies) to 100% (all the strategies) [mean scores will be reported]; (c) to indicate whether they have created their own sleep treatment materials, such as sleep diary, worksheet, or video (response options: no/not relevant/yes with option to describe) [frequencies will be reported]; (d) to indicate whether they deliver TranS-C as a standalone intervention or whether they integrate it with other interventions/topics (e.g., for mental health, medication, housing, vocational training) (response options: I always deliver the sleep treatment as a stand-alone intervention; I sometimes integrate the sleep treatment with other interventions or topics; I always integrate the sleep treatment with other interventions or topics; not applicable) [frequencies will be reported]; and (e) to report the number of sessions in which they use TranS-C concepts for each patient, on average [mean scores will be reported].

#### Benefits: perceived health benefits for patients

##### Primary outcome

The Outcomes & Effectiveness Scale is a 5-item scale from the Clinical Sustainability Assessment Tool [52], which assesses providers’ perceptions of TranS-C’s health benefits. Items are rated on a scale from 0 (to little or no extent) to 7 (to a very great extent)^4^. The mean score will be reported, and higher scores indicate more perceived benefits. This measure has demonstrated acceptable construct validity in similar contexts, and the subscale has demonstrated satisfactory internal consistency reliability (Cronbach’s alpha = 0.93) [52].

#### Capacity: knowledge, skills, and resources

##### Primary outcomes

The Skills Subscale from the Determinants of Implementation Behavior Questionnaire [53] assesses providers’ perceptions of their skills to deliver TranS-C. Three items are rated on a scale from 1 (strongly disagree) to 7 (strongly agree), where higher scores indicate more skills. The mean score will be reported. This subscale has demonstrated good discriminant validity and internal consistency reliability (Cronbach’s alpha = 0.86) [53, 54].

The Organizational Resources Subscale from the Implementation Potential Scales [55] is used to assess providers’ perceptions of whether they have the resources, support, and time needed to deliver TranS-C. Three items are rated on a scale from 1 (strongly disagree) to 6 (strongly agree), and the mean score is reported, where higher ratings indicate more perceived resources, support, and time. This subscale has demonstrated good construct validity and internal consistency reliability (Cronbach’s alpha = 0.85) in a sample of school psychologists [55].

##### Secondary outcomes

The Administrator Support Subscale from the Implementation Potential Scales [55] is used to assess the extent to which providers perceive they have support from leadership and supervisors to deliver TranS-C. Three items are rated on a scale from 1 (strongly disagree) to 6 (strongly agree), and the mean score is reported, where higher scores indicate more support. This subscale has demonstrated good construct validity and internal consistency reliability (Cronbach’s alpha = 0.86) in a sample of school psychologists [55].

Two knowledge items, modeled on Kauth et al. [40], are rated on a scale from 0 (not at all) to 7 (extremely), where higher scores indicate more knowledge. Specifically, providers rate the extent to which they (1) understand the theory and concepts behind TranS-C and (2) have the knowledge to conduct TranS-C. Mean scores of each item will be reported.

#### Proposed mechanism: provider perceptions of fit

Providers rate the acceptability, appropriateness, and feasibility of TranS-C via the following measures: Acceptability of Intervention Measure, Intervention Appropriateness Measure, and Feasibility of Intervention Measure [56]. Each of these measures consists of four items that are rated on a scale from 1 (completely disagree) to 5 (completely agree), where the mean of items is taken and higher scores indicate greater acceptability, appropriateness, and feasibility, respectively. These measures have demonstrated satisfactory validity and reliability in a convenience sample of mental health counselors [56].

#### Semi-structured interview

Providers are invited to complete a semi-structured interview that consists of 11 questions, each of which focuses on one of the following domains per Shediac-Rizkallah and Bone’s sustainment framework [12]: continued TranS-C activities, perceptions of TranS-C’s continued benefits to patients, and continued capacity to deliver TranS-C. Pre-specified as well as impromptu probes are used to assess possible mechanisms of outcomes. The first author (LDS) drafted the interview, then questions were refined with input from the facilitators, collaborators, and members of the research team. Following recommendations by implementation science experts [57], qualitative methods are included to gather more in-depth information about sustainment outcomes and mechanisms from the perspective of providers.

### Procedure

The sustainment surveys and interview are delivered one time at least three months after graduation to the Sustainment Phase (i.e., after the Sustainment Period). Before participating in this study, all providers give informed consent via secure, online forms (Docusign or Qualtrics) and are informed that they can withdraw from the study at any time. As noted above, providers are compensated according to their CMHC’s policy. Throughout all phases of the trial, providers are masked to treatment condition (i.e., Standard or Adapted TranS-C).

The surveys are compiled into a single assessment battery and administered on a version of Qualtrics that is compliant with the Health Insurance Portability and Accountability Act (HIPAA). Semi-structured interviews are delivered by the first author, members of the research team, and the lead facilitator via phone or HIPAA-compliant Zoom. The latter is used to record the interviews. Interviewers are not masked to treatment condition to enable asking appropriate probes during the interview. However, interviewers are thoroughly trained to deliver the interviews with integrity and minimal bias. The first author listens to interview recordings and provides feedback, and group/individual supervision is provided as needed.

### Allocation

During the Implementation Phase, CMHCs were randomized to Standard or Adapted TranS-C through a computerized randomization sequence by a biostatistician with no stratification at the CMHC or provider level. Throughout all phases of the trial, sites retain their original randomization assignment to Standard or Adapted TranS-C. Only the facilitators and research team (i.e., not CMHCs or providers) are privy to which CMHCs and providers are allocated to which TranS-C treatment condition (Standard TranS-C or Adapted TranS-C).

### Sample size

The number of providers for the quantitative analyses in this study (*N* = 154; 140 plus 10% for dropout) was selected based on recruitment from the Implementation Phase. Sample size determination was not needed for aim 1, which consists of descriptive statistics (see "Planned Analyses" section below). For aim 2, using this sample size in a cluster-randomized trial design, a minimum detectable effect size was calculated using Stata [58, 59]. Prior studies have reported moderate to large correlation coefficients between sustainment outcomes (*r*s = 0.34–0.64) [46, 60]. Based on the site intra-class correlation (ICC) estimated from similar prior studies [39, 61], the ICC was assumed to be 0.01. The coefficient of variation of cluster size was calculated as 0.31, based on the ratio of standard deviation of cluster size to mean cluster size [62]. A two-sided alpha of 0.05 was used. Together, the minimum detectable effect size using a cluster-randomized design with a sample of 140 across 10 clusters was a small to medium effect size of *d* = 0.40. Given that a prior study with a similar aim and outcomes produced a large effect size [63], we expect that it will be feasible to detect a small to medium effect size. For aim 3, a Monte Carlo power analysis through Schoemann et al.’s [64] application was conducted for parallel mediators with 1000 and 5000 replications, 20,000 Monte Carlo draws per replication, and 95% confidence intervals per recommendations. Drawing from prior research, large correlations (*r* = 0.50) were assumed between: the predictor (Standard vs. Adapted) and mediators (acceptability, appropriateness, feasibility) [63]; the mediators (*r* = 0.50) [56, 65]; and the mediators and sustainment outcomes (*r* = 0.50) [17–19]. Moderate correlations (*r* = 0.30) were assumed between the predictors and outcomes [19]. Given these estimates, the power detected for the indirect effects with a sample size of *N* = 140 was high (0.92–0.98).

For qualitative analyses, the target sample size (*N* = 40; *n* = 20 providers from Standard TranS-C, *n* = 20 providers from Adapted TranS-C) was guided by findings that saturation can be reached with an upper bound of 17 interviews [66].

### Data management and dissemination

All patient-identifiable data are saved on a secure password-protected and HIPAA-compliant website. After data have been collected, provider-identifiable data are removed and providers are assigned identification numbers. Participant-identifiable data are not shared with outside entities during or after the trial. The first author is responsible for downloading, collating, and analyzing the data.

A Data Safety Monitoring Board has been formed to help prevent and manage adverse events. The board includes members with expertise in SMI, psychosocial treatments, and randomized controlled trials. Members are independent from the first author (LDS), principal investigator (AGH), and competing interests. A report was made to the board bi-annually for the first year of the research conducted during the Implementation Phase (phase 1). Since then, the schedule has shifted to annual reports. However, if safety issues arise, the schedule will be changed to monthly reports. Yearly reports are submitted to the Committee for the Protection of Human Subjects at University of California, Berkeley, and the National Institute of Mental Health (NIMH). Triyearly reports on recruitment are also submitted to NIMH. Although TranS-C is a low-risk intervention, and there are no known negative effects, providers have been trained to alert their CMHC supervisor or the study team if negative effects are experienced by a patient. In such scenarios, the study team is available to work with the provider and CMHC staff to provide the patient with the appropriate supports and services. At the writing of this protocol, no adverse events have been reported across any phases of the trial.

During the Sustainment Phase, interim analyses are not conducted. Results from the trial, as well as analysis code, will be shared via peer-reviewed publications, professional conference presentations, and meetings and newsletters to CMHCs, as relevant and regardless of the magnitude/direction of effects. Authorship on future trial publications will be determined according to the guidelines set forth by the American Psychological Association [67]. Other than the authors and compliance with data-sharing agreements stipulated by the National Institutes of Health, no other entities have contractual agreements to access the final dataset. Deidentified data are submitted to the NIMH Data Archive twice per year, per NIMH requirements.

### Roles and responsibilities

This study is led by the first author (LDS), who supervises the Sustainment Phase research team and is responsible for data management, under the general supervision of the larger trial’s principal investigator (AGH), who also manages the facilitation team. The principal investigator and other collaborators offer expert guidance. The research team is responsible for the informed consent process, recruiting providers, and collecting data. The first author is responsible for downloading, collating, and analyzing the data. In addition, the first author regularly monitors recruitment and quality of data collected. The facilitators offer the minimal support to CMHCs during the Sustainment Phase, as described above. Members of all these teams will collaborate on writing up and disseminating the data. Communication occurs through as-needed meetings and regular email communication. There is no coordinating center, trial steering committee, Data Monitoring Committee (due to short timeframe and minimal risk), or Stakeholder and Public Involvement Group. The trial sponsor is University of California, Berkeley.^5^ Other than ethical approval for the study, the sponsor has no role or ultimate authority in study design; collection, management, analysis, or interpretation of the data; writing of the report; or the decision to submit the report for publication. With respect to audits, organizations not directly involved in the trial (e.g., NIMH, Committee for the Protection of Human Subjects, Data Safety Monitoring Board) have the right to audit and, if such a situation arises, will determine the frequency and procedures for auditing.

## Planned analyses

### Quantitative analyses

The present protocol describes all planned analyses for the primary aims of this study. Although subgroup and secondary analyses that are grounded in theory and/or empirical evidence may be conducted in the future, none have been preregistered or specified at the writing of this protocol. Because the first author played a key role in designing this study and will be responsible for leading data monitoring and analysis, the analyses will not be masked. However, to minimize potential bias, analyses will follow the prespecified plan described below.

#### Preliminary analyses and missing data

All analyses will use all available data (i.e., intent-to-treat, meaning all randomized participants, as randomized, who submitted any data for the Sustainment Phase will be included in the analyses) [68] via maximum likelihood estimation. Related assumptions regarding patterns of missingness (e.g., missing completely at random, missing at random, missing not at random) will be investigated by conducting Little’s MCAR test and testing the extent to which missingness is related to observed variables [69]. Baseline between-group differences in demographic variables will be examined and considered as possible covariates (e.g., depending on relationships to predictors and outcomes) [70]. Distributions will be evaluated to detect outliers, and we will ensure that the assumptions of planned analyses are met. ICCs will be reported. Data quality will be evaluated via range and mean checks.

#### Aim 1. Report TranS-C sustainment outcomes

Descriptive statistics of all primary and secondary sustainment outcomes (e.g., mean, standard deviation, range, frequency, percentage), as well as provider characteristics, will be reported.

#### Aim 2. Treatment condition on sustainment outcomes

Hierarchical linear modeling with maximum likelihood estimation will be used to test the effect of TranS-C condition (Standard vs. Adapted TranS-C) on primary sustainment outcomes, while accounting for providers (level 1) nested in CMHCs (level 2) [69]. The predictor will be represented by a dummy-coded variable for condition (1 = Adapted, with Standard as the reference group), and all outcomes will be modeled as continuous. The parameters of interest will be the effect of condition (Standard vs. Adapted TranS-C) on the primary sustainment outcomes.

#### Aim 3. Fit as a mediator of treatment condition and sustainment outcomes

Using structural equation modeling, multivariate parallel mediation models will test whether provider perceptions of acceptability, appropriateness, and feasibility mediate the relations between condition (dummy-coded as Adapted = 1, with Standard as the reference group) and primary sustainment outcomes (all continuous). Models will adjust for cluster (i.e., CMHCs). Three mediation models will be tested: one model will be evaluated for each category of sustainment outcomes (i.e., activities, benefits, capacity), and each model will simultaneously evaluate the three measures of fit (i.e., provider perceptions of acceptability, appropriateness, and feasibility).

#### Mixed methods analyses

Interviews will be coded and analyzed using thematic analysis [71] with a combination of deductive and inductive approaches [72], after which qualitative findings will be triangulated with survey data [73]. Interviews will be recorded and transcribed verbatim. The first author (LDS) will lead a coding team with expert input from other authors. Each coder will be required to establish 80% or higher inter-coder agreement with the first author across five interviews. The coding team (other than the first author) will be masked to provider condition (Standard vs. Adapted) and study hypotheses.

Deductive and inductive codebooks will be developed to guide data coding. The deductive codebook will consist of sustainment outcomes according to Shediac-Rizkallah and Bone’s sustainment framework (i.e., continued activities, benefits, and capacity) [12]. The first author will develop the inductive codebook by reading through all transcripts to become familiar with the data, then rereading the transcripts to identify inductive codes that emerge related to sustainment outcomes as well as possible predictors and mechanisms of sustainment outcomes (e.g., provider perceptions of fit) [71].

After the data have been deductively and inductively coded by the coding team, the first author will review the coded data, during which themes present in the interviews will be identified and refined [71, 72]. These themes will be used to analyze the extent to which sustainment outcomes were met, with respect to continued activities, benefits, and capacity (i.e., to supplement Specific Aim 1). Next, themes will be compared across the Standard and Adapted TranS-C conditions to help determine whether manipulating treatment fit to context impacts sustainment outcomes (i.e., to supplement Specific Aim 2). Additionally, themes will be analyzed to assess possible predictors and mechanisms of outcomes (i.e., to supplement Specific Aim 3). Triangulation will be conducted using a concurrent approach, in which interviews and surveys will be analyzed at the same time and given equal weight during interpretation [73]. Triangulation will be used to analyze the extent to which data converge, as well as to offer a deeper analysis of sustainment outcomes, predictors, and mechanisms [73, 74].

## Discussion

The present protocol describes the third and final phase—the Sustainment Phase—of a hybrid type 2 cluster-randomized controlled trial investigating the implementation and sustainment of the Transdiagnostic Intervention for Sleep and Circadian Dysfunction (TranS-C) for patients with serious mental illness (SMI) and sleep and circadian problems in community mental health centers (CMHCs). Research on the sustainment of evidence-based psychological treatments (EBPTs) in routine practice settings, such as CMHCs, is limited [9]. The present study seeks to advance our understanding of sustainment predictors, mechanisms, and outcomes by investigating (a) whether the implementation strategy of adapting an EBPT (i.e., TranS-C) to the CMHC context predicts improved sustainment outcomes and (b) whether this relation is mediated by improved provider perceptions of treatment fit. In turn, such findings may take steps toward supporting causal models of implementation and sustainment and inform more precise implementation efforts that effectively contribute to lasting change [15].

These potential contributions notwithstanding, several methodological limitations are important to consider for the Sustainment Phase. First, provider-level data are the focus, because providers are responsible for the day-to-day execution of EBPTs and are therefore essential to EBPT sustainment [75]. Unfortunately, it was not feasible to collect data at other levels (e.g., leadership, patients), given funding, timing, and partner priorities. Evaluating leadership- and patient-level predictors, mechanisms, and outcomes of sustainment in CMHCs will continue to be a critical direction for future research [76, 77]. Second, given the many demands on CMHC providers’ time, we carefully selected surveys that were relatively brief and straightforward. We strived to ensure that measures for all primary outcomes had published evidence to support adequate psychometric properties. However, some of the secondary measures consisted of unvalidated items that were based on prior research or derived for the present study (e.g., assessing the extent to which providers still use the provider manual and patient workbook), when we could not find brief, previously validated measures. Similarly, certain types of measures (e.g., behavioral assessments, knowledge tests, electronic health records) may have conferred advantages relative to self-report (e.g., assessing skills and knowledge or penetration) [78] but were not feasible to include in the present study. Third and related, readiness for sustainment is determined by the facilitation team. We considered utilizing an existing, validated measure to evaluate readiness for sustainment (e.g., Clinical Sustainability Assessment Tool) [52, 79], which might have standardized this process and eliminated some variability. However, we decided against delivering such measures, given the considerable number of surveys that community partners already complete for this trial. Instead, as described above, facilitators assess sustainment readiness across several different criteria and develop tailored sustainment plans that are completed with community partners, which concurrently serve to equip partners with a plan for sustainment and help facilitators assess readiness. Fourth, providers in the present study are eligible for sustainment data collection after their CMHC of employment has been in the Sustainment Phase for at least 3 months. Although prior research has suggested that clinics are at risk of sustainment failure as early as 3 months [42], further research will be needed to evaluate longer-term sustainment outcomes. Fifth, the COVID-19 pandemic and subsequent mandates (e.g., shelter-in-place)—which began in California shortly after Implementation Phase data collection began—introduced several challenges that may impact sustainment of TranS-C (e.g., rapid shift to virtual care; heightened focus on securing basic needs for patients; increased burnout) [80, 81]. This context should be considered when drawing implications from the findings. Despite these limitations, findings from the present study may provide empirical support for theoretical models of sustainment, support a possible roadmap toward EBPT sustainment in CMHCs, improve our understanding of treatment adaptation in routine practice settings, and meaningfully contribute to the clinical care of patients that is offered by invaluable providers.

## Trial status

Protocol version 1, September 5, 2023. Recruitment and data collection for the Sustainment Phase started in May 2023 and may continue through March 2024. Publishing of this protocol was delayed because of unforeseen challenges and uncertainties related to the COVID-19 pandemic and subsequent mandates (e.g., shelter-in-place), which began in California shortly after data collection started for the parent three-phase randomized controlled trial.

### Supplementary Information

**Additional file 1. **SPIRIT Checklist for Trials.

## Data Availability

Other than the authors and compliance with data-sharing agreements stipulated by the National Institutes of Health, no other entities have contractual agreements to access the final dataset. Deidentified data are submitted to the NIMH Data Archive twice per year, per their requirements.

## Footnotes

1 Note that the vast majority of providers were employed by CMHCs. However, some providers left their CMHC position during the study. Also, in very few isolated instances, providers outside of CMHCs learned about the study (e.g., by word of mouth) and requested to participate. When the latter providers otherwise met the criteria, they were permitted to participate and were matched with a CMHC patient by the facilitation team. For consistency across phases of the trial and to maximize sample size, these providers (i.e., who left their CMHC after receiving TranS-C training or requested to participate from outside of CMHCs) will be included in Sustainment Phase recruitment efforts.

2 The original measure used a 5-point scale from 0 to 4. However, this measure was delivered to providers using a 4-point scale at earlier timepoints in the present trial; thus, a 4-point scale was used for consistency.

3 In the original measure [50], the nightmares module was not included due to administrative error. It was added to the version administered to providers in Standard TranS-C (Adapted TranS-C does not include the nightmares module).

4 Due to administrative error, this scale deviates from the original scale of 1 to 7.

5 Contact information of the sponsor: +1(510) 642-6000; University Avenue and Oxford Street, Berkeley, CA 94702.
